# High-Affinity Nanobody Against the LEDGF PWWP Domain Inhibits Chromatin Binding In Vitro

**DOI:** 10.3390/biom16050716

**Published:** 2026-05-13

**Authors:** Thibault Vantieghem, Sofie Jansen, Thatcher Zinabu Akele, Pieterjan Van Maele, Sam Noppen, Dominique Schols, Maarten Dewilde, Zeger Debyser, Sergei V. Strelkov

**Affiliations:** 1 Biocrystallography, KU Leuven, 3000 Leuven, Belgium; t.vantieghem@lumc.nl (T.V.); sofie.jansen@kuleuven.be (S.J.); 2 Advanced Disease Modelling, Targeted Drug Discovery and Gene Therapy (ADVANTAGE), KU Leuven, 3000 Leuven, Belgium; thatcher.akele@kuleuven.be (T.Z.A.); zeger.debyser@kuleuven.be (Z.D.); 3 Laboratory for Therapeutic and Diagnostic Antibodies, KU Leuven, 3000 Leuven, Belgium; pieterjan.vanmaele@kuleuven.be (P.V.M.); maarten.dewilde@kuleuven.be (M.D.); 4 Molecular, Structural and Translational Virology, Rega Institute for Medical Research, KU Leuven, 3000 Leuven, Belgium; sam.noppen@kuleuven.be (S.N.); dominique.schols@kuleuven.be (D.S.); 5 PharmAbs-The KU Leuven Antibody Center, KU Leuven, 3000 Leuven, Belgium

**Keywords:** LEDGF/p75, PWWP domain, nanobody, single-domain antibody, VHH, chromatin reader, epigenetics, protein–protein interaction, binding study, inhibitor

## Abstract

**Background and objectives:** The PWWP domain of lens epithelium-derived growth factor p75 (LEDGF/p75) mediates chromatin engagement through recognition of histone H3 lysine 36 di- and trimethylation (H3K36me2/3) and nucleosomal DNA. LEDGF/p75 plays a role in multiple human diseases. In particular, its interaction with HIV-1 integrase enables viral genome integration. However, the LEDGF PWWP domain remains difficult to target with small molecules as it lacks optimally shaped binding pockets. Here, we report the generation of high-affinity nanobodies (Nbs) to investigate the structure and function of this domain. **Methods:** Camelids were immunized with recombinant LEDGF PWWP domain, and immune phage display libraries were screened for affinity. Selected Nbs were recombinantly expressed in *E. coli* and purified. Their interaction with the PWWP domain of LEDGF and its close homolog HRP-2 was characterized using size-exclusion chromatography and surface plasmon resonance. Structural characterization of the Nbs was performed using X-ray crystallography. Functional effects on chromatin engagement were evaluated using an AlphaScreen assay. **Results:** Nine sequence-distinct Nbs were identified, seven of which were confirmed to bind the LEDGF PWWP domain with nanomolar affinities. Five Nbs also bound the HRP-2 domain, consistent with conserved functional surfaces, while two showed reduced affinity. The crystal structures of two Nbs (NbC03 and NbH10) confirmed there were canonical immunoglobulin folds, while the latter additionally revealed a domain-swapped dimer. Moreover, NbH10 dose-dependently inhibited the interaction between full-length LEDGF/p75 and H3K36me3-modified nucleosomes in vitro. **Conclusions:** This work establishes a validated panel of Nbs targeting the LEDGF PWWP domain and identifies one Nb capable of functionally disrupting the LEDGF–chromatin interaction. These Nbs serve as valuable tools for functional studies and structure-based drug design.

## 1. Introduction

Lens epithelium-derived growth factor p75 (LEDGF/p75) is a chromatin-associated protein that plays a central role in transcriptional regulation and viral integration through its N-terminal PWWP domain. This domain mediates chromatin engagement by recognizing di- and trimethylated lysine 36 on histone H3 (H3K36me2/3) in conjunction with interactions with nucleosomal DNA. Beyond its physiological role, LEDGF/p75 is co-opted by HIV-1 integrase to direct viral genome integration into actively transcribed regions [1,2,3,4]. Dysregulation of LEDGF/p75 binding partners, such as MLL fusion proteins, has been implicated in oncogenic transcriptional programs [5,6]. As such, the LEDGF PWWP domain has emerged as a target of interest for epigenetic drug discovery [6,7].

Structural studies have shown that the LEDGF PWWP domain engages chromatin by recognizing H3K36me2/3-modified histones, engaging in specific interactions with the methylated lysine while also interacting with nucleosomal DNA [8,9,10]. This multivalent contact is critical for chromatin targeting [10,11,12]. Small-molecule ligands designed to target the H3K36me2/3 pocket typically exhibit only low micromolar affinity, and attempts to extend binding into adjacent regions have met with limited success [13]. Together, these observations highlight the need to use alternative molecular tools to probe the functional surfaces of the PWWP domain.

Single-domain antibodies, also known as nanobodies (Nbs), offer unique advantages as high-affinity molecular probes for challenging protein surfaces. Their comparatively small size, high solubility, and generally long complementarity-determining region 3 (CDR3) loops enable them to recognize concave, shallow, or cryptic epitopes that are often poorly tractable with respect to both conventional antibodies and small-molecule ligands [14,15,16,17]. Although Nbs are not generally suited to intracellular therapeutic delivery [18], they are valuable as structural chaperones, conformational stabilizers, and functional inhibitors in biochemical and cellular studies [17,19].

Here, we report on the generation and characterization of a panel of Nbs targeting the LEDGF PWWP domain. Using animal immunization followed by phage display selection, we identified a diverse set of Nbs that bind the LEDGF PWWP domain with nanomolar affinity. We also characterized their solution properties and cross-reactivity with the closely related HRP-2 PWWP domain. While co-crystal structures of Nb-PWWP complexes could not be obtained, one Nb was found to inhibit the interaction between full-length LEDGF/p75 and H3K36me3-modified nucleosomes in vitro. The developed Nbs are convenient tools for probing the LEDGF PWWP domain and provide a foundation for mechanistic functional studies and structure-guided ligand development.

## 2. Materials and Methods

### 2.1. Protein Constructs

The human LEDGF PWWP domain (UniProt: O75475, residues 1–110) was cloned in a pET16b vector encoding an N-terminal His_10_-tag. A Ser-to-Cys mutation at position 62 (S62C) was introduced using Quik-Change site-directed mutagenesis to enable site-specific biotinylation on the face opposite to the H3K36me2/3 pocket. Primers were manually designed (forward: 5′ TT CCT TAC **TGC** GAA AAT AAG GAA AAG TAT GGC AAA C 3′, reverse: 5′ TT ATT TTC **GCA** GTA AGG AAA TAT ATC CTT TGG TC 3′) and ordered from Integrated DNA Technologies (Leuven, Belgium).

A shorter LEDGF PWWP construct (residues 1–90) was cloned into a pETSUK2 vector encoding an N-terminal His_6_-tag followed by the small ubiquitin-like modifier (SUMO) domain, as described previously [20].

Human HRP-2 PWWP (UniProt: Q7Z4V5, residues 1–93) and its C64S mutant were prepared as described previously [13].

After Nb generation, selection, and sequencing (Section 2.4), nine representative Nb sequences were subcloned from the His_6_-FLAG_3_-tag vector into the pETSUK2 vector using the following primers: forward, 5′ GCGAACAGATTGGTGGTGGTGAGGTGCAATTGGTGGAGTCT 3′; and reverse, 5′ TTGTTAGCAGAAGCTTATTATGAGGAGACGGTGACCTGG 3′.

FLAG-LEDGF/p75 was expressed from pCp-NAT-FLAG_3_-LEDGF/p75 in *E. coli* BL21 Star (DE3) (Thermo Fisher Scientific, Waltham, MA, USA). Cultures were grown in LB medium containing 100 µg/mL of ampicillin at 37 °C until OD_600_ around 0.6, induced with 0.5 mM IPTG, and incubated for 3–4 h at 29 °C before harvesting.

All other constructs were overexpressed in heat-shock-transformed *E. coli* Rosetta^TM^ 2 (DE3) pLysS competent cells (Merck, Darmstadt, Germany) using auto-induction in ZYP-5052 medium [21,22]. Cells were harvested via centrifugation and stored at −80 °C.

### 2.2. Protein Purification

Unless otherwise stated, all chemicals were purchased from Merck. Most proteins were purified using a five-step protocol consisting of immobilized metal affinity chromatography (IMAC), tag cleavage, subtractive IMAC (sIMAC), ion-exchange chromatography (IEX), and size-exclusion chromatography (SEC). Specific buffer compositions used for each protein for IEX and SEC are listed in Table S1. For the LEDGF PWWP (residues 1–110) construct and the S62C mutant, the His_10_-tag was retained, and the sIMAC step was omitted. For HRP-2 PWWP and the LEDGF PWWP S62C mutant, 10 mM DTT was included in all IEX and SEC buffers. For Nb purification, reducing agents were omitted, and the IEX step was skipped; instead, Nb samples were dialyzed directly into the SEC buffer.

Cell pellets were thawed on ice and resuspended in low-imidazole buffer (50 mM sodium phosphate (pH 7.5), 250 mM NaCl, 12.5 mM imidazole (pH 7.5), and 5 mM β-mercaptoethanol), supplemented with SigmaFast protease inhibitor cocktail (1 tablet/100 mL) and Cryonase^TM^ Cold-Active Nuclease (1 U/mL, Takara Bio Europe SAS, Saint-Germain-en-Laye, France). Cells were lysed via sonication and clarified through centrifugation.

The supernatant was loaded onto a His-Select^®^ Nickel Affinity Gel resin (Merck) column pre-equilibrated with low-imidazole buffer. The column was subsequently washed with low-imidazole buffer supplemented with 0.1% *v*/*v* Triton^TM^ X-100, and the target protein was eluted with high-imidazole buffer (50 mM sodium phosphate (pH 7.5), 250 mM NaCl, 500 mM imidazole (pH 7.5), and 5 mM β-mercaptoethanol). For His_6_-SUMO-tagged constructs, tag cleavage was performed overnight with SUMO Hydrolase 7K (1:500) during dialysis (3 kDa) against low-imidazole buffer, followed by sIMAC to remove the cleaved tag. For constructs subjected to IEX, samples were dialyzed into a low-salt buffer supplemented with 10% *w*/*v* glycerol. All subsequent chromatographic steps were carried out using an Äkta^TM^ Pure system with a UV detector (GE Healthcare Europe, Diegem, Belgium). IEX was performed using HiTrap^®^ SP HP or Q HP columns (5 mL; Cytiva, Uppsala, Sweden), equilibrated in low-salt buffer, and proteins were eluted using a linear NaCl gradient with an increasing salt concentration. Protein fractions were pooled and then concentrated using Amicon^®^ Ultra filters (3 kDa cut-off) (Merck Millipore, Darmstadt, Germany). SEC was performed using a Superdex 75 16/60 GL column (GE Healthcare Europe) equilibrated with SEC buffer. Protein fractions were concentrated, flash-frozen in liquid nitrogen, and stored at −80 °C.

FLAG-LEDGF/p75 was purified using heparin affinity chromatography followed by SEC. Pellets were resuspended in 500 mM NaCl, 30 mM Tris-HCl (pH 7.4), 1 mM DTT, complete protease inhibitor cocktail (Roche, Basel, Switzerland), and DNase I (Roche) for lysis by sonication. The lysate was clarified via centrifugation and filtered through a 0.22 µm syringe filter before being loaded onto a 5 mL HiTrap Heparin HP column (Cytiva) equilibrated in 150 mM NaCl, 30 mM Tris-HCl (pH 7.0), and 1 mM DTT. Bound protein was eluted with a linear salt gradient from 150 mM to 2 M NaCl. Peak fractions were pooled and further purified by SEC on a Superose™ 6 10/300 GL column (Cytiva) equilibrated in 150 mM NaCl, 30 mM Tris-HCl (pH 7.4), and 1 mM DTT. Fractions containing FLAG-LEDGF/p75 were supplemented with 10% *v*/*v* glycerol, flash-frozen, and stored at −80 °C.

For phage display and the Nb selection AlphaScreen, the LEDGF PWWP S62C mutant was biotinylated using the EZ-Link^TM^ Maleimide Protein Labeling Kit (Thermo Fisher Scientific).

### 2.3. Immunization and Nanobody Library Preparation

One llama and one alpaca were immunized with recombinant LEDGF PWWP domain (residues 1–110). Animals received four injections in two-week intervals. Nb libraries were generated as described previously [23]. Peripheral blood mononuclear cells were isolated, total RNA was extracted, and first-strand cDNA was synthesized using oligo(dT) primers. Nb-encoding sequences were amplified via nested PCR and cloned into an in-house phagemid vector. Recombinant phagemids were transformed into *E. coli* TG1 cells, and Nb-displaying phages were produced using M13KO7 helper phage.

### 2.4. Nanobody Selection

Two rounds of phage display panning were performed using 100 nM biotinylated LEDGF PWWP. Streptavidin-coated Pierce™ beads (Thermo Fisher Scientific) were used in the first round, and Dynabeads™ (Thermo Fisher Scientific) were used in the second. Following enrichment, Nb sequences were subcloned into an expression vector encoding an OmpA signal peptide and a C-terminal FLAG_3_-His_6_ tag and expressed in *E. coli* TG1.

Periplasmic extracts were screened via AlphaScreen using 2 nM biotinylated LEDGF PWWP and anti-FLAG acceptor beads (20 µg/mL, PerkinElmer, Shelton, CT, USA). After incubation, streptavidin donor beads (PerkinElmer) were added, and signals were measured on an EnVision plate reader (PerkinElmer). Clones producing signals ≥3-fold above background were sequenced and clustered (≥91% identity), yielding nine clusters (Figure S1).

### 2.5. Binding Studies

#### 2.5.1. Size-Exclusion Chromatography

SEC binding studies were performed using a Superdex 200 10/300 GL column (GE Healthcare) equilibrated in 50 mM Tris-HCl (pH 7.0) and 150 mM NaCl. Each Nb was injected individually (2 mg/mL). LEDGF PWWP (residues 1–90) was injected separately at a concentration corresponding to a 1:1.1 molar ratio relative to the Nb. For complex analysis, LEDGF PWWP and the respective Nbs were pre-mixed at these final concentrations to maintain a 1:1.1 molar ratio.

#### 2.5.2. Surface Plasmon Resonance

Surface plasmon resonance (SPR) was used to characterize the molecular interaction between the Nbs and LEDGF using a Biacore^TM^ 8K instrument (Cytiva). LEDGF PWWP (residues 1–90) was covalently coupled on the surface of eight flow cells of a CM5 Series S sensor chip (Cytiva) using standard amine coupling and a running buffer composed of 10 mM HEPES (pH 7.4), 150 mM NaCl, 3 mM EDTA, and 0.05% Tween 20. First, carboxymethylated groups of the chip were activated for 7 min with a mixture of 200 mM 1-ethyl-3-(3-dimethylaminopropyl) carbodiimide hydrochloride (EDC) and 50 mM N-hydroxysuccinimide (NHS) at 10 µL/min. Then, 2 µg/mL of LEDGF PWWP diluted in 10 mM HEPES (pH 7) was injected for 6 min at 10 µL/min. The remaining activated groups were blocked with 1 M ethanolamine-HCl (pH 8.5) for 7 min at 10 µL/min. The obtained LEDGF PWWP immobilization levels were about 200 RU. Eight reference flow cells without LEDGF PWWP were used as a control for non-specific binding and refractive index changes (i.e., one reference flow cell per LEDGF PWWP flow cell). All binding experiments were performed at 25 °C in 50 mM Tris-HCl (pH 7.4) supplemented with 150 mM NaCl and 0.05% Tween 20. Three-fold serial dilutions (12.3, 37.0, 111.1, 333.3, and 1000 nM) of Nbs were sequentially injected from low to high concentrations for 120 s at a flow rate of 30 μL/min in a single cycle. The dissociation was monitored for 15 min, and finally the surface was regenerated with 50 mM NaOH. Several buffer blanks were used for double referencing. The equilibrium dissociation constant (K_D_) was calculated after fitting the experimental data to the 1:1 Langmuir interaction model using Biacore Insight Evaluation Software (version 6.0). All SPR experiments were performed in triplicate.

The interaction between the Nbs and the HRP-2 PWWP domain was analyzed using the same protocol, with immobilization levels ranging from 300 to 800 RU.

### 2.6. Protein Crystallization

Initial crystallization screening of the Nbs and their complexes with LEDGF PWWP (1–90) or HRP-2 PWWP C64S mutant (prepared in 1:1 molar ratio) was carried out using commercial crystallization kits (Molecular Dimensions, Hampton Research, Qiagen, and Rigaku) at 4 °C and 20 °C. Screening was performed using the sitting-drop method in Swissci 96-Well 2-drop plates (Hampton Research, Aliso Viejo, CA, USA), with 75 µL of reservoir solution per well. We set up two drops per condition, consisting of either 150 nL + 150 nL or 200 nL + 100 nL of protein and crystallization solution, respectively, by using a Mosquito robot (SPT Labtech, Hertfordshire, UK). The individual protein concentrations ranged from 5 to 12 mg/mL. For crystallization of the complexes, the Nb and the LEDGF PWWP domain or the HRP-2 PWWP C64S mutant domain were mixed in a 1:1 molar ratio at a low concentration, incubated to allow complex formation, and then concentrated together to the desired final concentration. Plates were sealed, stored, and imaged using Rock Imager (Formulatrix, Bedford, MA, USA).

Optimizations of crystallization hits were performed using either the sitting-drop method with the same setup as in the initial screening or the hanging-drop vapor diffusion method in 24-well XRL^TM^ plates (Molecular Dimensions, Rotherham, UK). Hanging-drop crystallization involved 2 µL drops and 500 µL reservoir solution. NbC03 crystals were grown at 20 °C via sitting-drop vapor diffusion. Drops consisted of 150 nL of protein solution (6.7 mg/mL) + 150 nL of crystallization solution (1.5 M ammonium sulfate, 12% glycerol and 0.1 M Tris-HCl pH 8.5). NbH10 crystals were grown at 20 °C via sitting-drop vapor diffusion. NbH10 and LEDGF PWWP domain were mixed in a 1:1 molar ratio and concentrated (12 mg/mL of NbH10 and 10 mg/mL of LEDGF PWWP—final concentrations). Drops consisted of 200 nL of protein mixture + 100 nL of crystallization solution (1 M succinic acid, 1% PEG MME 2000, and 0.1 M HEPES (pH 7.0)).

### 2.7. X-Ray Crystallography

Crystals were harvested, cryoprotected using crystallization solution supplemented with 10–20% ethylene glycol, and cryo-cooled in liquid nitrogen. X-ray data were collected at beamlines ID30A-3 [24] and ID30B [25] of the European Synchrotron Radiation Facility (ESRF) at 100 K using an X-ray wavelength close to 1 Å. Data were processed using autoPROC with the STARANISO option [26,27] and phased via molecular replacement using PHASER [28] and AlphaFold3 models of the Nbs [29]. Structures were fitted in COOT [30], refined in REFMAC5 [31] and Phenix [32], and analyzed using PyMOL (Version 3.0, Schrödinger, LLC, New York, NY, USA). Crystallographic statistics are given in Table S2. Crystal interfaces were analyzed using PISA software version 1.52 [33].

### 2.8. AlphaFold3 Modeling of the Nanobody–LEDGF PWWP Complexes

The LEDGF PWWP domain and Nb sequences were submitted as paired inputs to the AlphaFold3 web server [29] to predict the structure of the 1:1 complexes. Modeling was performed using default parameters, and the resulting models were evaluated using the interface predicted template modeling (ipTM) score to assess the confidence of the predicted binding interface.

### 2.9. AlphaScreen Inhibition Assay

AlphaScreen in vitro inhibition assays were performed in a 384-well OptiPlate (PerkinElmer) with a final volume of 25 µL. Nbs were diluted in SEC buffer, while FLAG-LEDGF/p75 and reagents were diluted in AlphaScreen buffer (25 mM Tris-HCl (pH 7.4), 150 mM NaCl, 1 mM MgCl_2_, 1 mM DTT, 0.1% Tween 20, and 0.1% bovine serum albumin). FLAG-LEDGF/p75 and varying Nb concentrations were pre-incubated for 30 min at 4 °C; this was followed by addition of H3K36me3-modified nucleosomes and incubation for 1 h at 4 °C. Optimal concentrations determined by cross-titration were 10 nM FLAG-LEDGF/p75 and 5 nM nucleosomes. Donor and acceptor beads (10 µg/mL each, PerkinElmer) were then added, and plates were incubated for 1h at room temperature before being read on an Envision Xcite Multilabel Reader (PerkinElmer). Counts were plotted, and a non-linear regression sigmoidal curve fit was obtained using GraphPad Prism 10.6.1 (San Diego, CA, USA).

## 3. Results and Discussion

### 3.1. Generation and Selection of LEDGF PWWP-Specific Nanobodies

One llama and one alpaca were immunized with the recombinant human LEDGF PWWP domain, and immune phage display libraries were generated from VHH repertoires. Following two rounds of panning against the antigen, 94 individual clones were screened for binding to the human LEDGF PWWP domain using periplasmic extracts in an AlphaScreen assay. Twenty-eight clones produced signals that were at least three-fold above the background. Sequencing yielded twelve distinct Nb sequences. Clustering analysis (with a ≥91% sequence identity cut-off) revealed one cluster comprising three sequences (NbA03, NbB03 and NbG01) differing mainly in their CDRs, another cluster comprising two sequences (NbA02 and NbC02) that differ in one framework residue, and seven further singlets (Figure S1) [34].

### 3.2. Expression, Purification, and Solution Behavior of Selected Nanobodies

One representative Nb from each cluster (Figure S1) was selected for large-scale expression as a His_6_-SUMO fusion protein in the cytoplasm of *E. coli*. Even though recombinant expression of Nbs has traditionally been targeted towards periplasm with the aim of facilitating proper disulfide formation in oxidative conditions, more recent data suggest that cytoplasmic expression may also be used successfully and with higher yields [35]. After expression, NbA03 exhibited reduced solubility and partial precipitation after tag removal and was excluded from further analysis. The remaining eight Nbs were expressed in soluble form and purified using sIMAC with tag cleavage (Figure S2A).

All Nbs were further purified by SEC using the Superdex 75 16/60 GL column (Figure S3). The SEC profiles, with the exception of NbB11, NbG08, and NbH10, revealed the tallest, symmetric elution peak at around 80 mL, presumably corresponding to a monomer. For NbC08, additional left-hand peaks were present. Judging by SDS-PAGE, these peaks still contained the target Nb, suggesting the presence of higher oligomeric species. For NbB11, the dominant major peak was at 120 mL, preceded by minor peaks between 40 and 50 mL, which corresponded to impurities. For NbG08, two sharp peaks at 67 and 93 mL were observed, both corresponding to the Nb. They were assumed to represent oligomeric (possibly dimeric) and monomeric species, respectively. As the presumed monomeric peak of NbG08 precipitated after SEC, the higher-oligomer fraction was used for subsequent experiments. For NbH10, three main peaks were observed. The first two peaks at 45 and 75 mL contained NbH10 in association with impurities, whereas the third peak at 110 mL corresponded to pure NbH10, as confirmed by SDS-PAGE (Figure S2B,C).

### 3.3. High-Affinity Binding of Nanobodies to the LEDGF PWWP Domain

**Figure 1:**
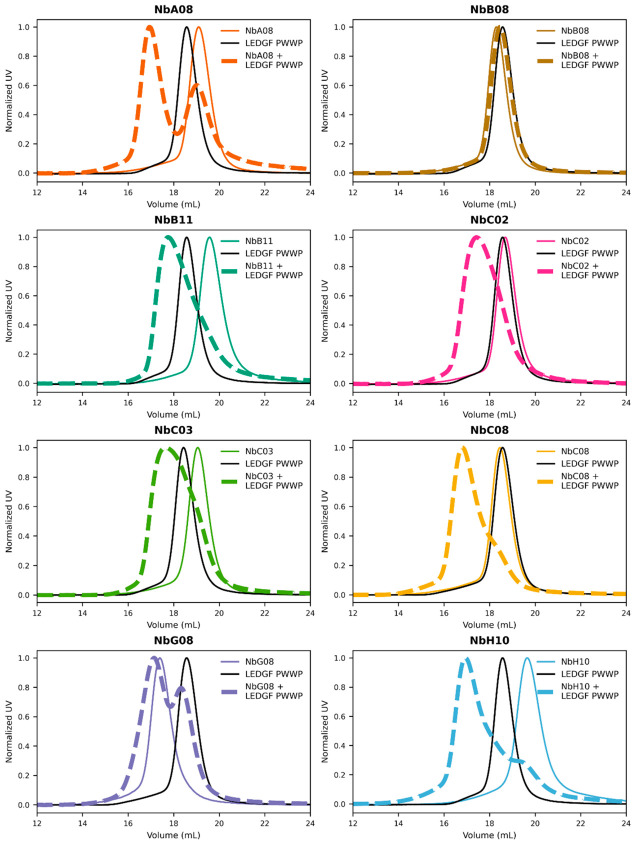
Size-exclusion chromatography on Superdex 200 10 × 300 GL column for each nanobody, LEDGF PWWP, and their 1.1:1 molar mixes. The profiles were normalized by the main peak height.

**Figure 2:**
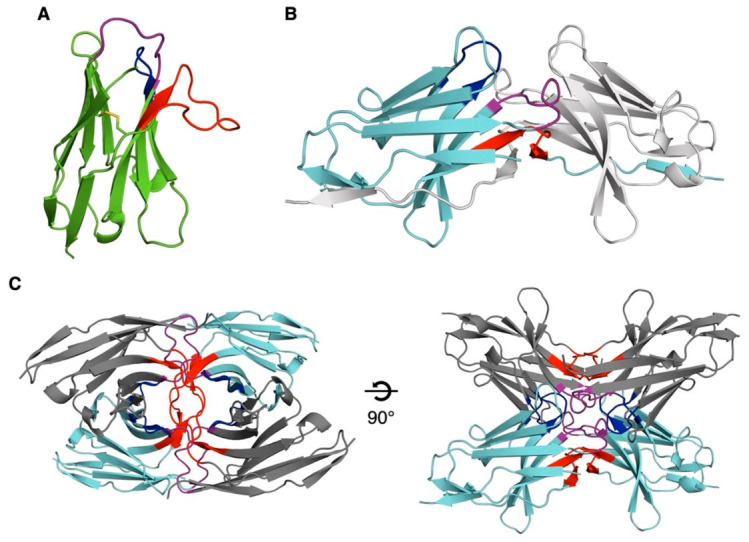
Crystal structures of NbC03 and NbH10 shown as ribbon diagrams. CDR1, CDR2, and CDR3 are shown in purple, blue, and red, respectively. (**A**). A single NbC03 molecule is shown, with the disulfide bridge between Cys22 and Cys96 depicted as sticks. (**B**). Two neighboring NbH10 molecules interact through their CDR1 (purple) and CDR2 (blue) loops. Their CDR3 loops and the final β-strand are exchanged, resulting in a domain-swapped dimer. (**C**). Two NbH10 domain-swapped dimers (cyan and dark grey) interact with each other, resulting in a tetrameric NbH10 assembly inside the crystal.

All SEC-purified Nb samples were used in an initial test of binding to the LEDGF PWWP domain by means of analytical SEC (Figure 1). To this end, each Nb, the LEDGF PWWP domain, and the Nb:LEDGF PWWP domain mixture in a 1.1:1 molar ratio were applied to a Superdex 200 10 × 300 GL column. The LEDGF PWWP domain eluted at ca. 18.5 mL. The individual Nbs A08, B08, C02, C03 and C08 eluted as sharp single peaks at 18.5–19 mL, closely matching the elution volume of the PWWP domain. As the hydrodynamic radii of the LEDGF PWWP domain and the Nbs (exemplified by the crystal structure of NbC03—see Figure 2A) are very similar (1.78 and 1.85 nm, respectively, as calculated using HullRad [36]), the comparable elution behavior confirms that these Nbs are present in a monomeric state. NbB11 and NbH10 displayed a slightly delayed peak at ca. 20 mL, which could be caused by interaction with the column. Finally, the NbG08 sample eluted at 17.5 mL, i.e., earlier than the other individual Nbs, supporting the notion that this Nb was present as an oligomer (see above).

Importantly, for all Nb:LEDGF PWWP domain mixtures except for NbB08, the SEC profiles readily revealed the emergence of a new left-shifted, tallest peak at 16.5–17.5 mL elution, which was indicative of complex formation (Figure 1). A rather similar position of this peak across all Nbs (including NbG08) suggested that all of them yield a 1:1 complex with the PWWP domain. In addition, smaller lower-molecular weight peaks or ‘tailing’ were observed, which pointed to the presence of some free components. In contrast, NbB08 did not exhibit any detectable complex formation under these conditions.

As the next step, the binding affinities were determined through SPR experiments with immobilized LEDGF PWWP and Nbs as analytes. To this end, SEC-purified Nb samples were used, except for NbH10, which was only purified through sIMAC, still yielding >90% purity (Figure S2C).

In agreement with the SEC-based interaction studies, NbB08 showed no measurable binding. All seven remaining Nbs bound to LEDGF PWWP with apparent K_D_ values ranging from 46 to 292 nM when fitted using a 1:1 interaction model (Table 1 and Figure S4). Some deviations from the theoretical fits at higher concentrations were observed for NbB11, NbC02, and NbH10, which may be explained by secondary, less specific binding events.

Of note, while NbA08 and NbG08 had two-digit nanomolar apparent affinities in SPR, both Nbs revealed partial complex dissociation in SEC (Figure 1). Such behavior is not unprecedented for protein complexes. Indeed, the SPR experiments reveal the affinity between the immobilized PWWP domain and the Nb. In contrast, the SEC elution profile depends on several factors, including not only the K_D_ value of the complex but also its dynamic nature. Consequently, the association-dissociation equilibrium between the complex and the individual components, when occurring in the SEC flow, could yield a distinct result compared to the SPR measurement.

### 3.4. Cross-Reactivity with the HRP-2 PWWP Domain

We additionally studied the interaction of the Nbs with the PWWP domain of hepatoma-derived growth factor-related protein 2 (HRP-2), which is a paralog of LEDGF. While the two domains have an overall sequence identity of 79%, their H3K36me2/3 pockets and surroundings are nearly identical (Figure S5). Our SPR experiments revealed that NbB11, NbC03, NbC08, NbG08 and NbH10 bound HRP-2 PWWP with affinities close to those observed for LEDGF PWWP (Table 1). We therefore hypothesize that all these Nbs bind to the conserved regions (Figure S5). Given that the H3K36me2/3 pocket and its borders correspond to a major conserved patch, it is highly plausible that these Nbs are binding at or near the pocket, but additional data are needed for confirmation.

In contrast, NbA08 and NbC02 showed substantially reduced affinities for HRP-2, suggesting they bind to LEDGF-specific surface features such as those present outside the conserved H3K36me2/3 pocket and nucleosomal DNA binding surface (Figure S5). Of note, given that NbA08 and NbC02 recognize distinct epitopes between the two paralogs, these Nbs may be suitable for applications requiring LEDGF selectivity.

### 3.5. Structural Characterization of Nanobodies

Extensive crystallization trials were performed for the eight purified Nbs alone, for equimolar mixes with the LEDGF PWWP domain, and for equimolar mixtures with HRP-2 PWWP. In two cases, namely, NbC03 and the NbH10-LEDGF PWWP mixture, diffraction quality crystals were obtained (Table S2).

Crystals of NbC03 diffracted to 2.9 Å resolution. The asymmetric unit contained two Nb molecules, each adopting a canonical immunoglobulin fold stabilized by a conserved disulfide bond between Cys22 and Cys96 (Figure 2A), which was well resolved in an electron density map. The CDR1, CDR2, and CDR3 loops comprise eight, seven, and fourteen residues, respectively, confirming correct folding and a typical Nb architecture.

Crystals grown from the NbH10-LEDGF PWWP mixture diffracted to 2.3 Å resolution. However, structure determination revealed the presence of NbH10 alone. Two NbH10 molecules were present per asymmetric unit, each forming a domain-swapped dimer through exchange of the CDR3 loop and terminal β-strand via a two-fold crystallographic symmetry axis (Figure 2B). Domain swapping has previously been reported for Nbs, both as a crystallization artifact [37]—often promoted by high protein concentrations, ionic strength, or short CDR3 loops—and, in some cases, as a functionally relevant assembly capable of antigen binding [38]. The software product PISA [33] identified large interfaces both within the domain-swapped dimer and between the two chains in the asymmetric unit. The latter interface is mediated by both CDR1 and CDR2. All interfaces were predicted to be energetically favorable (Table S3). Collectively, these inter-subunit interactions yield a highly stable tetramer with 222 symmetry (Figure 2C).

Given the lack of co-crystal structures, Nb-PWWP complexes were also modeled using AlphaFold3. However, all the models generated exhibited low interface confidence scores (ipTM scores < 0.3), consistent with the current limitations of structure prediction for antigen-recognizing protein–protein interfaces lacking strong co-evolutionary signals [39].

### 3.6. NbH10 Inhibits the LEDGF/p75-H3K36me3 Nucleosome Interaction In Vitro

**Figure 3:**
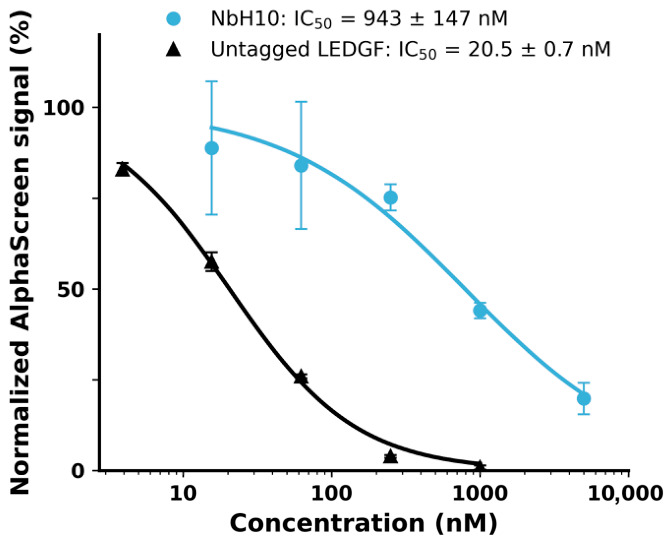
NbH10 dose-dependently inhibits the LEDGF/p75-nucleosome interaction. AlphaScreen assays were performed using 10 nM FLAG-LEDGF/p75 and 5 nM biotinylated H3K36me3 nucleosome. Increasing concentrations of NbH10 or untagged LEDGF/p75 were added to compete with FLAG-LEDGF/p75 for nucleosome binding. The normalized AlphaScreen signal (%) is plotted against inhibitor concentration on a logarithmic scale. Curves represent nonlinear regression fits (four-parameter logistic). Data points show means ± standard deviations of three independent experiments.

The functional impact of Nb binding was assessed using an AlphaScreen assay measuring the interaction between the full-length LEDGF/p75 and H3K36me3-modified nucleosomes. Here, NbH10 produced a dose-dependent inhibition of this interaction with an IC_50_ of 0.94 ± 0.15 μM (Figure 3). This demonstrates that NbH10 engages a functionally relevant surface of the PWWP domain involved in chromatin recognition. Given that this interface is fully conserved between LEDGF and HRP-2, this observation is well in line with the very similar affinities of ~100 nM measured for this Nb against both the LEDGF and HRP-2 PWWP domains (Table 1). None of the other Nbs, including NbA08, inhibited the interaction between LEDGF/p75 and the nucleosomes (Figure S6). Notably, NbA08 binds the isolated LEDGF PWWP domain with the highest affinity (46 nM) but shows approximately 100-fold lower affinity for the HRP-2 domain (Table 1), suggesting that it recognizes a distinct, non-functional surface rather than the nucleosome-binding site (Figure S5).

## 4. Conclusions

This study presents the first systematic generation and characterization of Nbs targeting the PWWP domain of LEDGF/p75. We show that as many as seven of the identified Nbs bind the isolated PWWP domain, with an affinity ranging between 46 and 292 nM, and display paralog-selective properties. This chromatin reader domain is therefore readily targetable by Nbs, thus overcoming the previously encountered challenges with developing high-affinity small-molecule ligands. Moreover, we proved that NbH10 is a functional, submicromolar inhibitor of the LEDGF/p75–H3K36me3 nucleosome interaction in vitro, which is also corroborated by its high binding affinity to both the LEDGF and HRP-2 PWWP domains.

Of note, our results indicate that Nbs isolated from the cytoplasm (rather than the periplasm) of *E. coli* can be correctly folded and functional. In particular, the established crystal structures of NbC03 and NbH10 unequivocally reveal the formation of an internal disulfide bond between the conserved pair of cysteine residues.

In summary, our study establishes a proof of principle that Nbs can interfere with PWWP-mediated chromatin engagement. In a broader sense, the Nb panel described here can be used as molecular tools for probing the LEDGF function. These results offer a foundation for future epitope-mapping, structural, and cellular studies targeting PWWP-dependent disease mechanisms.

## Figures and Tables

**Table 1:** SPR-based affinity values of nanobodies for LEDGF and HRP-2 PWWP domains.

	NbA08	NbB08	NbB11	NbC02	NbC03	NbC08	NbG08	NbH10
**LEDGF**	46.3 ± 3.5	No binding	112 ± 5	292 ± 21	116 ± 1	201 ± 20	71.7 ± 5.5	94.4 ± 9.1
**HRP-2**	4721 ± 1347	No binding	170 ± 5	1130 ± 150	100 ± 33	111 ± 28	98.7 ± 6.3	81.2 ± 1.8

All apparent dissociation constants (K_D_) are in nM and represent means ± standard deviations of triplicate measurements. “No binding” indicates no measurable interaction.

## Data Availability

X-ray diffraction data and atomic coordinates for NbC03 and NbH10 were deposited in the Protein Data Bank under the accession code 9TZZ and 9U00, respectively.

## Supplementary Materials

The following supporting information can be downloaded at https://www.mdpi.com/article/10.3390/biom16050716/s1. Figure S1: Alignment of unique nanobody sequences identified and clustered. Figure S2: SDS-PAGE for nanobody purification. Figure S3: SEC purification profiles of nanobody samples. Figure S4: Representative SPR sensorgrams and theoretical binding profiles of the nanobodies over immobilized LEDGF PWWP and HRP-2 PWWP. Figure S5: Comparison of the PWWP domain sequences. Figure S6: Inhibition of LEDGF/p75-nucleosome interaction by nanobodies measured via AlphaScreen. Table S1: Buffers used for ion-exchange chromatography and size-exclusion chromatography of different protein constructs. Table S2: Crystallographic statistics. Table S3: Crystal interface analysis regarding the NbH10 crystals.

## Abbreviations

The following abbreviations are used in this manuscript:

H3K36me2/3	Di-or trimethylated Lys36 of histone H3
IEX	Ion-exchange chromatography
IMAC	Immobilized metal affinity chromatography
K_D_	Dissociation constant
LEDGF/p75	Lens epithelium-derived growth factor p75
Nbs	Nanobodies
SEC	Size-exclusion chromatography
sIMAC	Subtractive IMAC
SPR	Surface plasmon resonance
SUMO	Small ubiquitin-like modifier

## References

[1] Cherepanov P., Ambrosio A.L.B., Rahman S., Ellenberger T., Engelman A. (2005). Structural Basis for the Recognition between HIV-1 Integrase and Transcriptional Coactivator P75. Proc. Natl. Acad. Sci. USA.

[2] Llano M., Saenz D.T., Meehan A., Wongthida P., Peretz M., Walker W.H., Teo W., Poeschla E.M. (2006). An Essential Role for LEDGF/P75 in HIV Integration. Science.

[3] Busschots K., Voet A., De Maeyer M., Rain J.-C., Emiliani S., Benarous R., Desender L., Debyser Z., Christ F. (2007). Identification of the LEDGF/P75 Binding Site in HIV-1 Integrase. J. Mol. Biol..

[4] Engelman A., Cherepanov P. (2008). The Lentiviral Integrase Binding Protein LEDGF/P75 and HIV-1 Replication. PLoS Pathog..

[5] El Ashkar S., Schwaller J., Pieters T., Goossens S., Demeulemeester J., Christ F., Van Belle S., Juge S., Boeckx N., Engelman A. (2018). LEDGF/P75 Is Dispensable for Hematopoiesis but Essential for MLL-Rearranged Leukemogenesis. Blood.

[6] Blokken J., De Rijck J., Christ F., Debyser Z. (2017). Protein–Protein and Protein–Chromatin Interactions of LEDGF/P75 as Novel Drug Targets. Drug Discov. Today Technol..

[7] Cermakova K., Weydert C., Christ F., De Rijck J., Debyser Z. (2016). Lessons Learned: HIV Points the Way Towards Precision Treatment of Mixed-Lineage Leukemia. Trends Pharmacol. Sci..

[8] Wu H., Zeng H., Lam R., Tempel W., Amaya M.F., Xu C., Dombrovski L., Qiu W., Wang Y., Min J. (2011). Structural and Histone Binding Ability Characterizations of Human PWWP Domains. PLoS ONE.

[9] Wang H., Farnung L., Dienemann C., Cramer P. (2020). Structure of H3K36-Methylated Nucleosome–PWWP Complex Reveals Multivalent Cross-Gyre Binding. Nat. Struct. Mol. Biol..

[10] Koutná E., Lux V., Kouba T., Škerlová J., Nováček J., Srb P., Hexnerová R., Šváchová H., Kukačka Z., Novák P. (2023). Multivalency of Nucleosome Recognition by LEDGF. Nucleic Acids Res..

[11] Eidahl J.O., Crowe B.L., North J.A., McKee C.J., Shkriabai N., Feng L., Plumb M., Graham R.L., Gorelick R.J., Hess S. (2013). Structural Basis for High-Affinity Binding of LEDGF PWWP to Mononucleosomes. Nucleic Acids Res..

[12] van Nuland R., van Schaik F.M., Simonis M., van Heesch S., Cuppen E., Boelens R., Timmers H.M., van Ingen H. (2013). Nucleosomal DNA Binding Drives the Recognition of H3K36-Methylated Nucleosomes by the PSIP1-PWWP Domain. Epigenetics Chromatin.

[13] Vantieghem T., Aslam N.A., Osipov E.M., Akele M., Van Belle S., Beelen S., Drexler M., Paulovcakova T., Lux V., Fearon D. (2024). Rational Fragment-Based Design of Compounds Targeting the PWWP Domain of the HRP Family. Eur. J. Med. Chem..

[14] Hamers-Casterman C., Atarhouch T., Muyldermans S., Robinson G., Hammers C., Songa E.B., Bendahman N., Hammers R. (1993). Naturally Occurring Antibodies Devoid of Light Chains. Nature.

[15] De Genst E., Silence K., Decanniere K., Conrath K., Loris R., Kinne J., Muyldermans S., Wyns L. (2006). Molecular Basis for the Preferential Cleft Recognition by Dromedary Heavy-Chain Antibodies. Proc. Natl. Acad. Sci. USA.

[16] Muyldermans S. (2013). Nanobodies: Natural Single-Domain Antibodies. Annu. Rev. Biochem..

[17] Pardon E., Laeremans T., Triest S., Rasmussen S.G.F., Wohlkönig A., Ruf A., Muyldermans S., Hol W.G.J., Kobilka B.K., Steyaert J. (2014). A General Protocol for the Generation of Nanobodies for Structural Biology. Nat. Protoc..

[18] Silva-Pilipich N., Smerdou C., Vanrell L. (2021). A Small Virus to Deliver Small Antibodies: New Targeted Therapies Based on AAV Delivery of Nanobodies. Microorganisms.

[19] Dingus J.G., Tang J.C., Amamoto R., Wallick G.K., Cepko C.L. (2022). A General Approach for Stabilizing Nanobodies for Intracellular Expression. eLife.

[20] Weeks S.D., Drinker M., Loll P.J. (2007). Ligation Independent Cloning Vectors for Expression of SUMO Fusions. Protein Expr. Purif..

[21] Studier F.W. (2005). Protein Production by Auto-Induction in High-Density Shaking Cultures. Prot. Expr. Purif..

[22] Studier F.W. (2014). Stable Expression Clones and Auto-Induction for Protein Production in *E. coli*. Methods Mol. Biol..

[23] Wouters Y., Jaspers T., De Strooper B., Dewilde M. (2020). Identification and In Vivo Characterization of a Brain-Penetrating Nanobody. Fluids Barriers CNS.

[24] Von Stetten D., Carpentier P., Flot D., Beteva A., Caserotto H., Dobias F., Guijarro M., Giraud T., Lentini M., McSweeney S. (2020). ID30A-3 (MASSIF-3)—A Beamline for Macromolecular Crystallography at the ESRF with a Small Intense Beam. J. Synchrotron Rad..

[25] McCarthy A.A., Barrett R., Beteva A., Caserotto H., Dobias F., Felisaz F., Giraud T., Guijarro M., Janocha R., Khadrouche A. (2018). ID30B—A Versatile Beamline for Macromolecular Crystallography Experiments at the ESRF. J. Synchrotron Rad..

[26] Vonrhein C., Flensburg C., Keller P., Sharff A., Smart O., Paciorek W., Womack T., Bricogne G. (2011). Data Processing and Analysis with the autoPROC Toolbox. Acta Crystallogr. D Biol. Crystallogr..

[27] Vonrhein C., Tickle I.J., Flensburg C., Keller P., Paciorek W., Sharff A., Bricogne G. (2018). Advances in Automated Data Analysis and Processing within *autoPROC*, Combined with Improved Characterisation, Mitigation and Visualisation of the Anisotropy of Diffraction Limits Using *STARANISO*. Acta Crystallogr. A Found. Adv..

[28] McCoy A.J. (2007). Solving Structures of Protein Complexes by Molecular Replacement with *Phaser*. Acta Crystallogr. D Biol. Crystallogr..

[29] Abramson J., Adler J., Dunger J., Evans R., Green T., Pritzel A., Ronneberger O., Willmore L., Ballard A.J., Bambrick J. (2024). Accurate Structure Prediction of Biomolecular Interactions with AlphaFold 3. Nature.

[30] Emsley P., Cowtan K. (2004). Coot: Model-Building Tools for Molecular Graphics. Acta Crystallogr. D Biol. Crystallogr..

[31] Murshudov G.N., Skubák P., Lebedev A.A., Pannu N.S., Steiner R.A., Nicholls R.A., Winn M.D., Long F., Vagin A.A. (2011). REFMAC5 for the Refinement of Macromolecular Crystal Structures. Acta Crystallogr. D Biol. Crystallogr..

[32] Liebschner D., Afonine P.V., Baker M.L., Bunkóczi G., Chen V.B., Croll T.I., Hintze B., Hung L.-W., Jain S., McCoy A.J. (2019). Macromolecular Structure Determination Using X-Rays, Neutrons and Electrons: Recent Developments in Phenix. Acta Crystallogr. D Struct. Biol..

[33] Krissinel E., Henrick K. (2007). Inference of Macromolecular Assemblies from Crystalline State. J. Mol. Biol..

[34] Lefranc M.-P., Pommié C., Ruiz M., Giudicelli V., Foulquier E., Truong L., Thouvenin-Contet V., Lefranc G. (2003). IMGT Unique Numbering for Immunoglobulin and T Cell Receptor Variable Domains and Ig Superfamily V-like Domains. Dev. Comp. Immunol..

[35] De Marco A. (2020). Recombinant Expression of Nanobodies and Nanobody-Derived Immunoreagents. Protein Expr. Purif..

[36] Fleming P.J., Fleming K.G. (2018). HullRad: Fast Calculations of Folded and Disordered Protein and Nucleic Acid Hydrodynamic Properties. Biophys. J..

[37] Spinelli S., Desmyter A., Frenken L., Verrips T., Tegoni M., Cambillau C. (2004). Domain Swapping of a Llama VHH Domain Builds a Crystal-wide Β-sheet Structure. FEBS Lett..

[38] Gallant J.P., Hicks D., Shi K., Moeller N.H., Hoppe B., Lake E.W., Baehr C., Pravetoni M., Aihara H., LeBeau A.M. (2024). Identification and Biophysical Characterization of a Novel Domain-Swapped Camelid Antibody Specific for Fentanyl. J. Biol. Chem..

[39] McCoy K.M., Ackerman M.E., Grigoryan G. (2024). A Comparison of Antibody–Antigen Complex Sequence-to-structure Prediction Methods and Their Systematic Biases. Protein Sci..

