# Supplementary material for: High-Affinity Nanobody Against the LEDGF PWWP Domain Inhibits Chromatin Binding In Vitro
Source: Biomolecules. 2026 May 13;16(5):716. doi: 10.3390/biom16050716 (PMC13204740; doi:10.3390/biom16050716)
Supplement: Supplementary file 1 [file biomolecules-16-00716-s001.zip › biomolecules-4168736-supplementary.pdf]

biomolecules

```
NbB08   1 EVQLVESGGGLVQPGGSLRLSCAASGNTLSINTMAWYRQ-ATGKQRELVATIRRDG-TKDYADSV 63

NbA08   1 EVQLVESGGGLVQPGGSLRLSCAASGFTFSRYAMSWVRQ-APGVELEWVSAINSGGSRTRYANSV 64

NbG01   1 EVQLVESGGGLVRAGRSLRLSCAASGSSFRIAAMGWYRR-TPGNQREFVALITSDG-VTNYGDSM 63
NbA03   1 EVQLVESGGGLVQAGRSLRLSCAASGSSFRIAAMGWYRR-PPGKQREFVALITSGG-TTNYGDSM 63
NbB03   1 EVQLVESGGGLVQAGRSLRLSCAASGSSFRIATMGWYRG-APGKQREFVALITSGG-STNYGDSM 63

NbA02   1 EVQLVESGGGLVQSGGSLRLSCAASGTIFTIHAMGWYRR-VPGKQRELVAVITNDG-NTNYADSV 63
NbC02   1 EVQLVESGGGLVQSGGSLRLSCAASGTIFTIHAMGWYRR-VPGKQRELVAVITNDG-NTNYADSV 63

NbC08   1 EVQLVESGGGLVQAGGSLRLSCAASGSIFGIDAMGWYRQ-APGNQRELVAVISRDG-SSNYADSV 63

NbC03   1 EVQLVESGGGLVQAGGSLRLSCAASGTIFSINAMGWYRRQAPGKQRELVAVITRGG-STNYADSV 64

NbG08   1 EVQLVESGGGFVQAGGSLRLSCAASGSIFSINAMGWYRR-APGNQRELVAVITSGG-RTNYADSV 63

NbB11   1 EVQLVESGGGLVQAGGSLRLSCAASGSIFSINAMGWYRR-VPGKQRELVAVISSGG-RTNYADSV 63

NbH10   1 EVQLVESGGGLVQAGGSLRLSCAASGSTFSIDAMGWYRR-AAGKQRELVAVISSGG-FTNYADSV 63

          **********:*: * **********  :   :*.* *    * : * *: *  .*  . *.:*:


NbB08  64 KGRFTISRDYAKNQMDLQMNSLEPGDTAVYYCNAEGLWSGGIERVRGPQSKYERWGQGTQVTVSS 128

NbA08  65 KGRFTISRDNAKNTLYLQMNSLKPEDTAVYYCAKGEP------DYYSDKWESDYWGQGTQVTVSS 123

NbG01  64 AGRCTISRDNAEKTIYLQVDSLKPEDTAVYYCVAGSI------R----SSRPDYWGQGTQVTVSS 118
NbA03  64 KGRFTISRDNAEKTIYLQIDSLKPEDTAVYYCVAGSI------H----SSRPDYWGQGTQVTVSS 118
NbB03  64 KGRFTISRDNAEKTIYLQIDSLKPEDTAVYYCVAGSI------R----SSRPDYWGQGTQVTVSS 118

NbA02  64 KGRFTISRDNAKNTVYLQMNSLKPEDTSVYYCTTDPH------RP---TADLRYWGQGTRVTVSS 119
NbC02  64 KGRFTISRDNAKNTVYLQMNSLKPEDTSVYYCTTDPH------RP---TADLRYWGQGTQVTVSS 119

NbC08  64 KGRFTISIDNLKNTVYLQMNSLRPEDTAVYYCAAGPI-------VV--LTRPRYWGQGTQVTVSS 119

NbC03  65 KGRFTISIDNAKNTVYLQMNSLKPEDTALYYCAADWA------VVT--TTPPRYWGQGTQVTVSS 121

NbG08  64 KGRFTISRDNAKNTVYLQMNSLKPEDTAVYFCAADER------VVV--IDRPRYWGQGTQVTVSS 121

NbB11  64 KGRFTISRDNAKNTVYLQMNSLKPEDSTVYYCAAGR-------------SYPDYWGQGTQVTVSS 115

NbH10  64 KGRFTISRDNAKNTVYLQMNSLKPEDTNVYYCTAGSN------------YYPNYWGQGTQVTVSS 116

           ** *** *  :: : **::**.* *: :*:*                     *****:*****
```

Figure S1. Alignment of unique nanobody sequences identified and clustered. Nanobodies shown in bold were expressed. CDR1, CDR2, and CDR3 were assigned using the IMGT system [1] and are highlighted in purple, blue and red, respectively. Residue numbering is sequential and consistent with the numbering used in the PDB structures. Mutations distinguishing members within the same cluster are highlighted in bold.

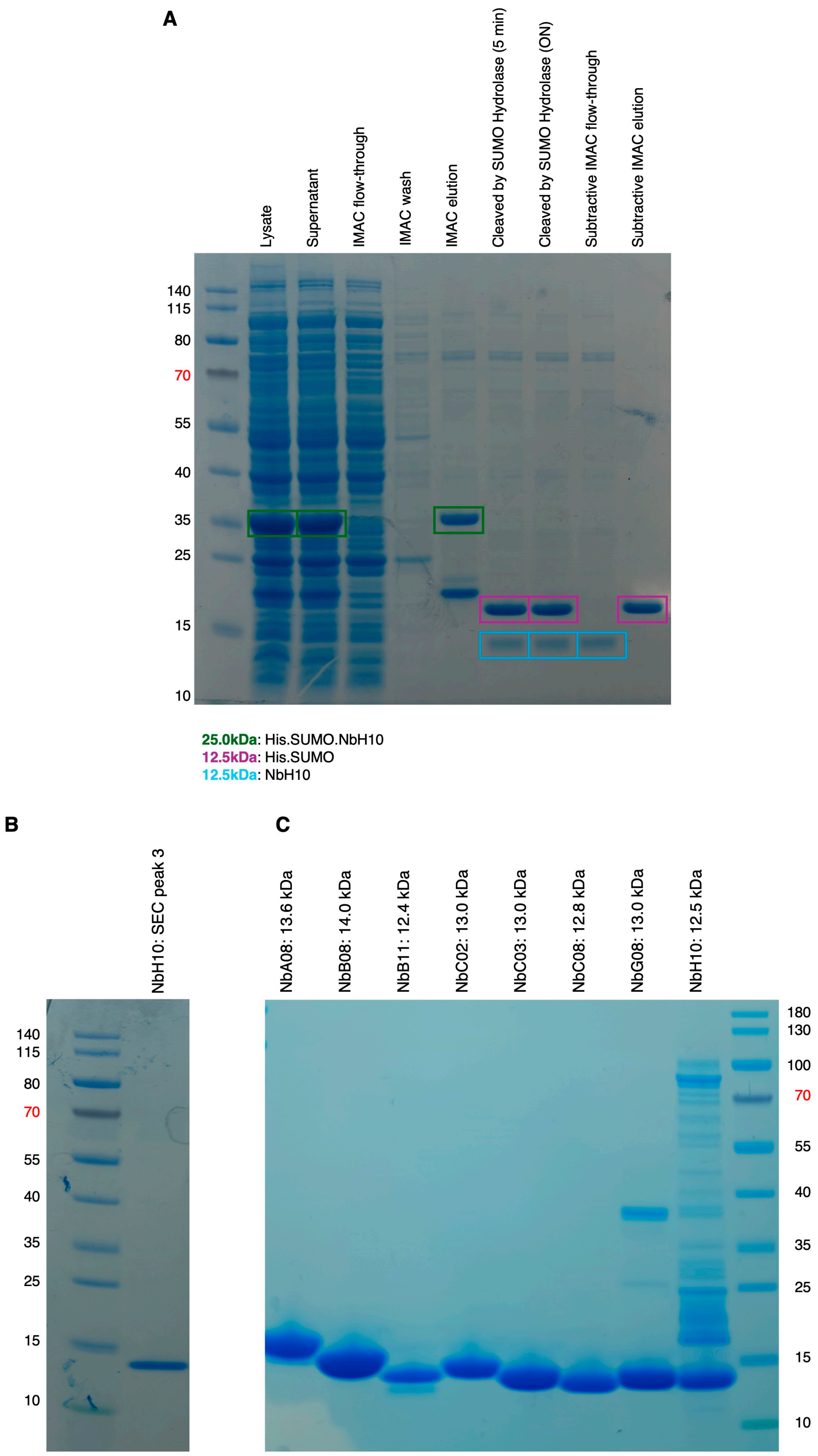

Figure S2. SDS-PAGE for nanobody purification. (**A**) SDS-PAGE of NbH10 IMAC and subtractive IMAC purification. (**B**) SDS-PAGE of SEC-purified NbH10 sample (Figure S3, peak 3 indicated by an asterisk) used for the SEC-based binding assay. (**C**) SDS-PAGE of the final nanobody samples used in the downstream assays including SPR-based binding to the PWWP domain, crystallography, and AlphaScreen, with their respective molecular weights. All samples were obtained with SEC as shown in Figure S3, except for NbH10 which was after subtractive IMAC.

https://doi.org/10.3390/biom16050716

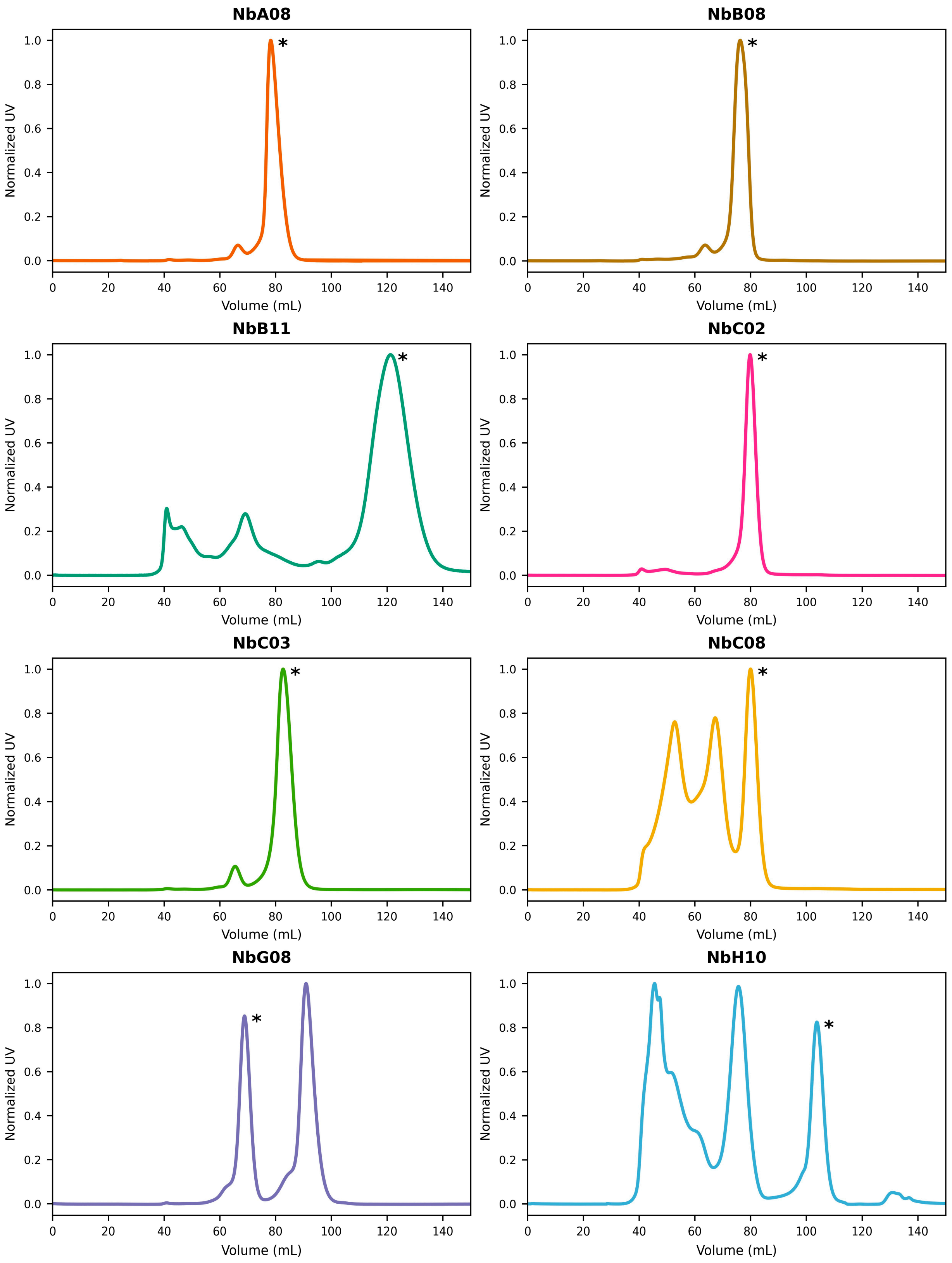

Figure S3. SEC purification profiles of nanobody samples**.** Size-exclusion chromatography profiles for eight nanobodies obtained on a Superdex 75 16/60 GL (V = 120 mL) column. The profiles were normalized by the main peak height. The peak fraction used for downstream analyses is indicated by an asterisk. For all nanobodies except NbG08, this corresponds to the presumed monomeric peak. For NbG08, the higher-oligomer fraction was used. For NbH10, the indicated fraction was used only for the SEC-based assay.

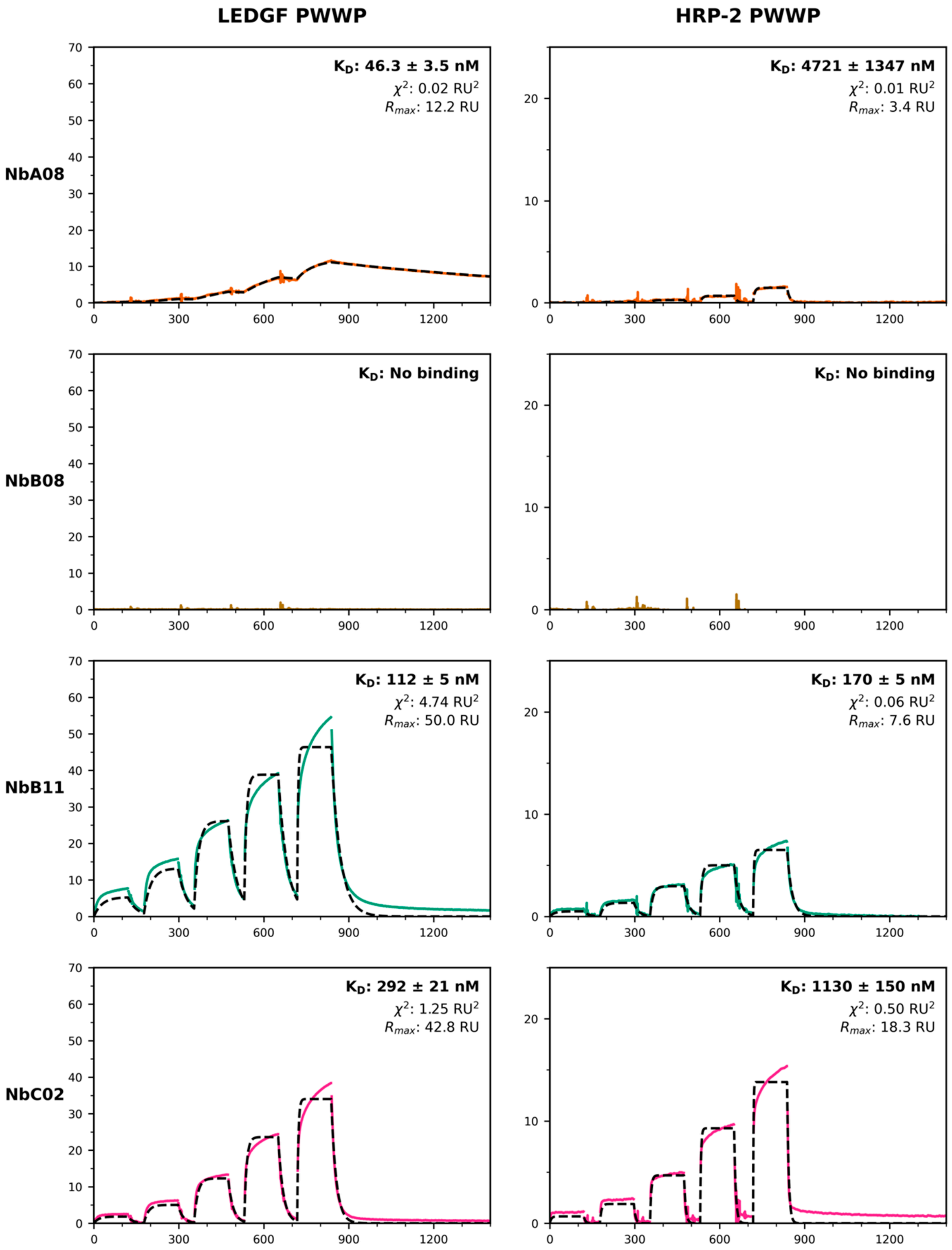

Figure S4. Representative SPR sensorgrams and theoretical binding profiles of the nanobodies over immobilized LEDGF PWWP and HRP-2 PWWP. Sequentially injected concentrations were 12.3, 37.0, 111.1, 333.3, and 1000 nM, except for NbA08 and NbC02 over HRP-2 PWWP, which were injected at 37.0, 111.1, 333.3, 1000, and 3000 nM. The apparent dissociation constant (K_D) for each interaction is indicated, with standard deviations from triplicate measurements. The goodness-of-fit (χ^2) for each sensorgram is reported.

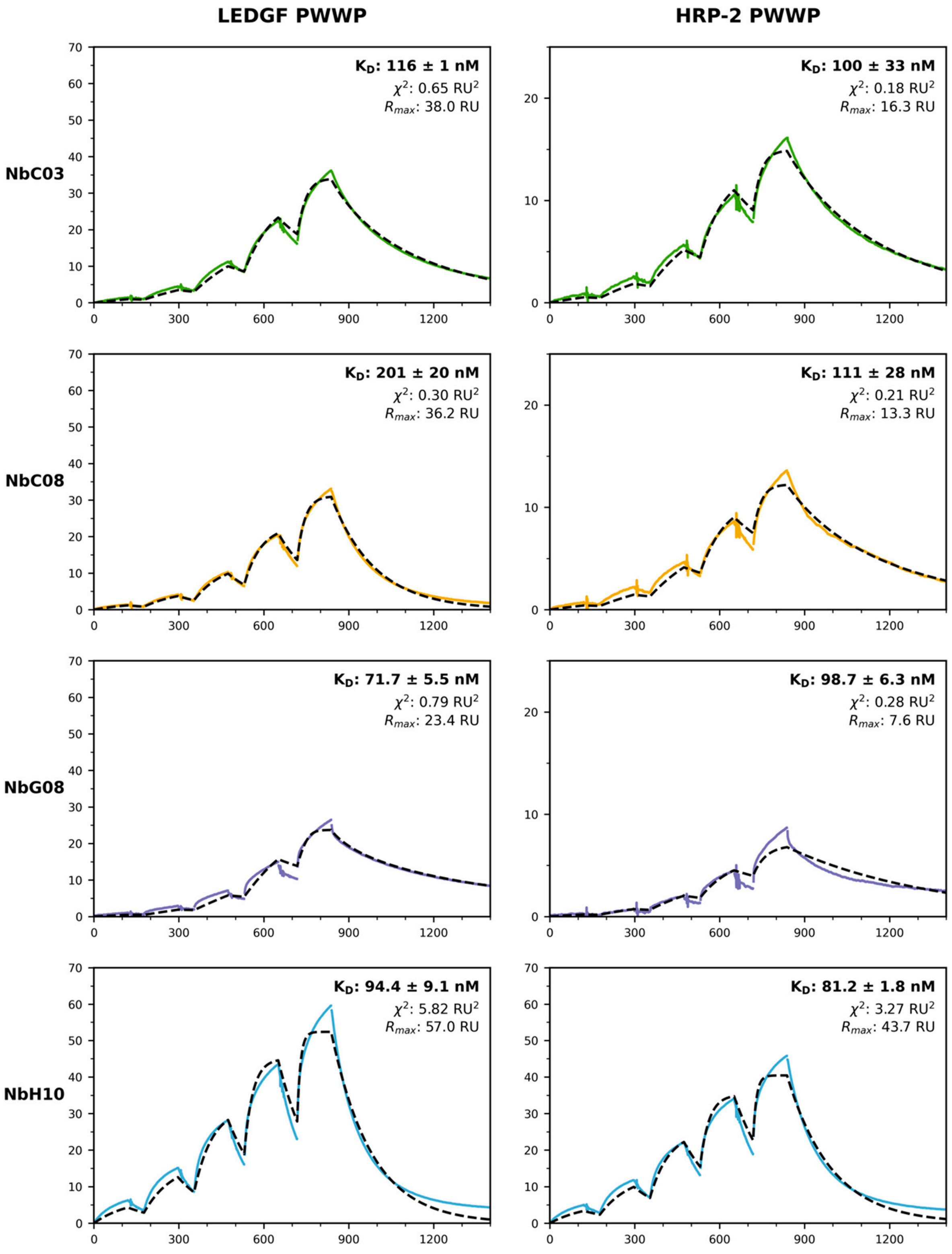

Figure S4. (continued).

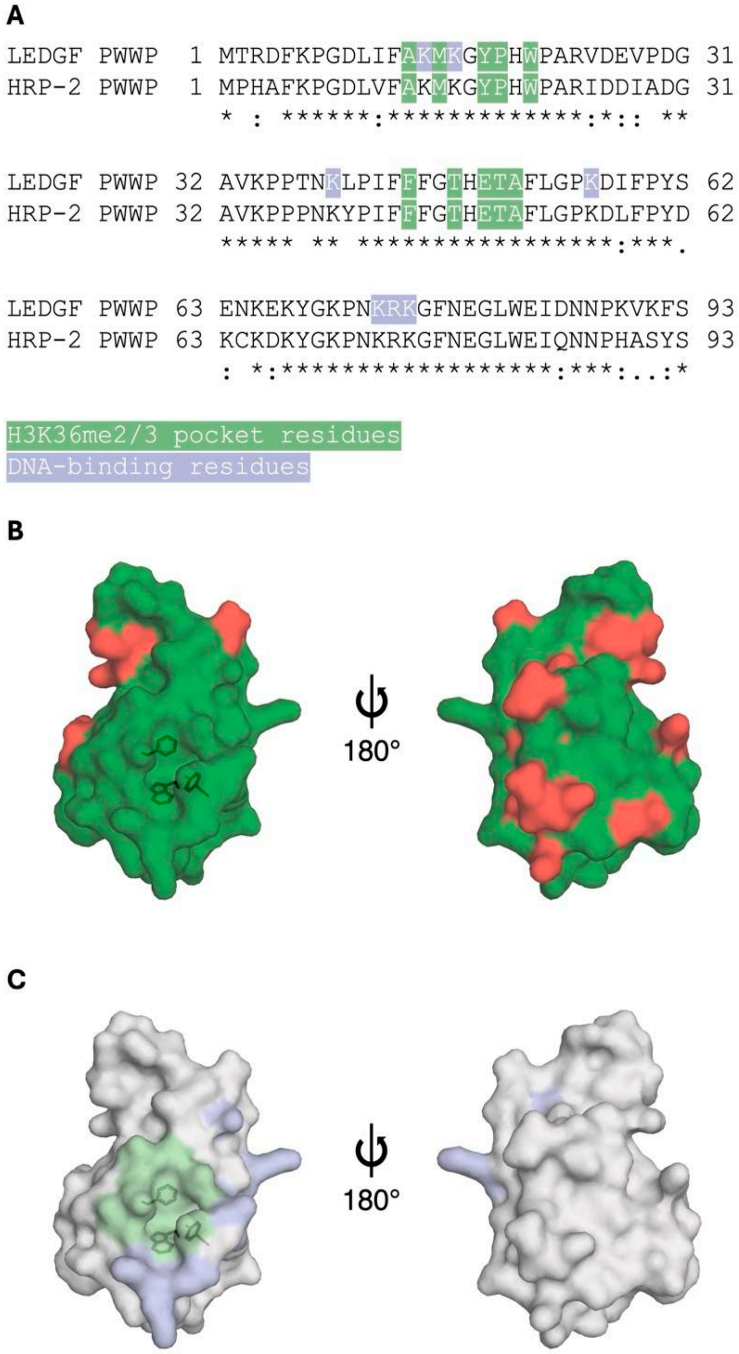

Figure S5. Comparison of the PWWP domain sequences. **A.** Sequence alignment with H3K36me2/3 pocket residues highlighted in green. LEDGF PWWP DNA-binding residues are highlighted in purple. The alignment was created with Clustal Omega. **B.** LEDGF PWWP domain surface colored by sequence identity with HRP-2. Dark green residues are identical and red residues are different. H3K36me2/3 pocket residues Tyr18, Trp21, and Phe44 are shown as sticks. **C.** LEDGF PWWP domain surface residues colored as in panel A.

https://doi.org/10.3390/biom16050716

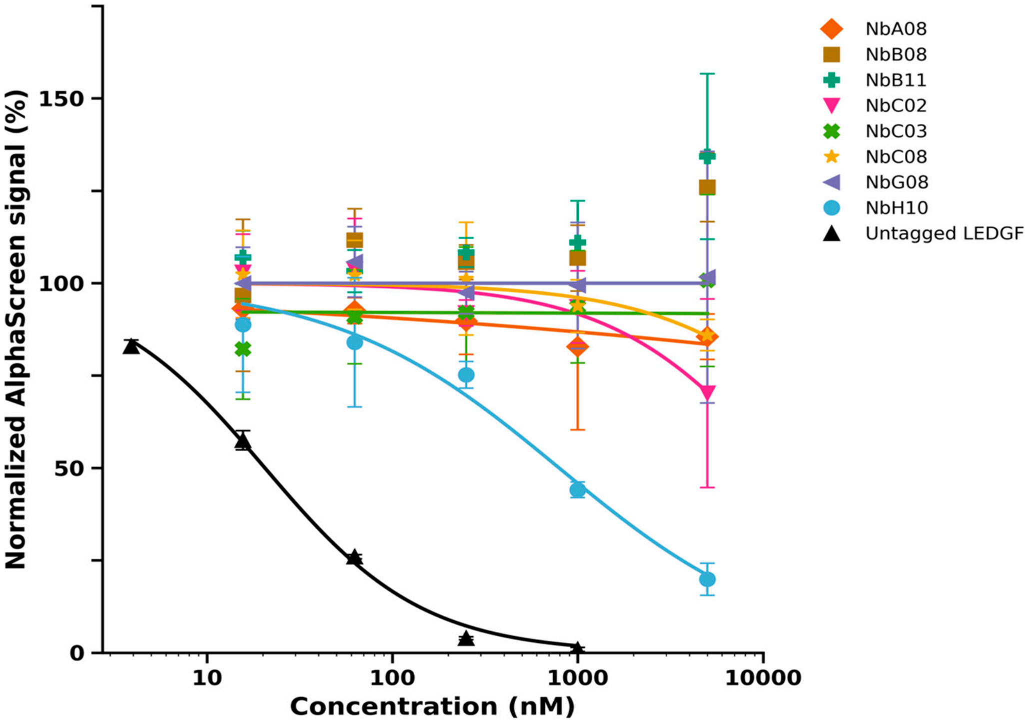

Figure S6. Inhibition of LEDGF/p75-nucleosome interaction by nanobodies measured via AlphaScreen. The normalized AlphaScreen signal (%) is plotted against inhibitor concentration on a logarithmic scale. Curves represent nonlinear regression fits (four-parameter logistic). Data points show mean ± standard deviation of three independent experiments.

Table S1. Buffers used for ion-exchange chromatography and size-exclusion chromatography of different protein constructs.

Protein construct	Predicted pI	Low-salt IEX buffer	SEC buffer
LEDGF PWWP (1-110)	8.8	50 mM HEPES pH 7.4	50 mM HEPES pH 7.4, 150 mM NaCl
LEDGF PWWP (1-110.S62C)	8.7	50 mM HEPES pH 7.4, 5 mM DTT	50 mM HEPES pH 8.0, 150 mM NaCl, 5 mM DTT
LEDGF PWWP (1-90)	9.2	50 mM HEPES pH 8.0	50 mM Tris-HCl pH 8.0, 150 mM NaCl
HRP-2 PWWP (1-93)	9.2	50 mM HEPES pH 8.0, 5 mM DTT	20 mM Tris-HCl pH 7.9, 150 mM NaCl, 5 mM DTT
HRP-2 PWWP (1-93.C64S)	9.3	50 mM HEPES pH 8.0	20 mM Tris-HCl pH 7.9, 150 mM NaCl
Nanobodies*	Variable	-	50 mM Tris-HCl pH 7.0, 150 mM NaCl
FLAG-LEDGF/p75‡	-	-	30 mM Tris-HCl pH 7.4, 150 mM NaCl, 1 mM DTT

* Ion-exchange chromatography was not performed for the nanobodies. ‡ Purified by heparin affinity chromatography and size-exclusion chromatography.

https://doi.org/10.3390/biom16050716

Table S2. Crystallographic statistics.

	NbC03<br>PDB entry 9TZZ	NbH10<br>PDB entry 9U00
**Crystallization**		
**Method**	Sitting drop	Sitting drop
**Temperature (°C)**	20	20
**Protein concentration (mg/mL)**	6.7	NbH10: 12.0<br>LEDGF PWWP: 10.0
**Protein buffer**	50 mM Tris-HCl pH 7.0<br>150 mM NaCl	50 mM Tris-HCl pH 7.0<br>150 mM NaCl
**Precipitant solution**	1.5 M Ammonium sulfate<br>12% Glycerol<br>0.1 M Tris-HCl pH 8.5	1 M Succinic acid<br>1% PEG MME 2000<br>0.1 M HEPES pH 7.0
**Volume and ratio protein:precipitant**	300 nL – 1:1	300 nL – 2:1
**Data collection & processing**		
**Diffraction source**	ESRF ID30B	ESRF ID30A-3
**Wavelength (Å)**	0.873	0.968
**Temperature (K)**	100	100
**Detector**	Dectris Eiger2 X 9M	Dectris Eiger1 X 4M
**Space group**	$P4_32_12$	$P6_522$
**a, b, c (Å)**	72.7; 72.7; 109.6	53.9; 53.9; 348.5
**α, β, γ (°)**	90; 90; 90	90; 90; 120
**Mosaicity (°)**	0.20	0.20
**Resolution range (Å)**	60.57 – 2.93 (3.69 – 2.93)	58.06 – 2.32 (2.65 – 2.32)
**No. of unique reflections**	5940 (2495)	9032 (481)
**Completeness (%)**	91.5 (46.2)	89.5 (54.0)
**Redundancy**	24.2 (27.5)	36.5 (34.6)
**Mean(I)/σ(I)**	4.0 (1.3)	12.2 (1.7)
**$CC_{1/2}$**	0.822 (0.493)	0.988 (0.623)
**Rmeas**	0.268 (0.683)	0.312 (3.680)
**Wilson B-factor (Å^2)**	24.85	37.09
**Structure solution & refinement**		
**Final R_{work}**	0.265 (0.299)	0.268 (0.401)
**Final R_{free}**	0.322 (0.336)	0.320 (0.463)
**No. protein chains per ASU**	2	2
**No. of non-H atoms**	1820	1708
Protein	1793	1708
Ligands	20	-
Solvent	7	-
**RMSD bonds (Å)**	0.002	0.003
**RMSD angles (°)**	0.47	0.55
**Average B-factor (Å^2)**	17.12	36.28
Protein	17.06	36.28
Ligands	23.18	-
Solvent	15.04	-
**Ramachandran favored (%)**	95.80	91.19
**Ramachandran allowed (%)**	3.36	6.17
**Ramachandran outlier (%)**	0.84	2.64

https://doi.org/10.3390/biom16050716

Table S3. Crystal interface analysis for the NbH10 crystals.

Interface	Chain 1	Chain 2	Interaction type	Interface area (Å²)	ΔG (kcal/mol)	No. H-bonds	CSS
1.1	A	A′	CDR3 domain swapping	2276	-24.9	54	1.000
1.2	B	B′	Same	2194	-25.3	44	1.000
2	A	B	CDR1 and CDR2	727	-11.8	2	1.000

A′ and B′ indicate symmetry mates of chain A and B, respectively. Chain A forms a domain-swapped dimer with A′. Chain B forms a similar domain-swapped dimer with B′. The complex significance score (CSS) ranges from 0 (unlikely to be biologically relevant) to 1 (highly likely).

https://doi.org/10.3390/biom16050716

References

1. Lefranc, M.-P.; Pommié, C.; Ruiz, M.; Giudicelli, V.; Foulquier, E.; Truong, L.; Thouvenin-Contet, V.; Lefranc, G. IMGT Unique Numbering for Immunoglobulin and T Cell Receptor Variable Domains and Ig Superfamily V-like Domains. *Developmental & Comparative Immunology* **2003**, *27*, 55–77, doi:10.1016/S0145-305X(02)00039-3.

Disclaimer/Publisher's Note: The statements, opinions and data contained in all publications are solely those of the individual author(s) and contributor(s) and not of MDPI and/or the editor(s). MDPI and/or the editor(s) disclaim responsibility for any injury to people or property resulting from any ideas, methods, instructions or products referred to in the content.
